# List of Reviewers

**DOI:** 10.1002/pcn5.70035

**Published:** 2024-11-28

The Editorial Board members of Psychiatry and Clinical Neurosciences Reports are grateful to the following reviewers who provided the Journal with their expertise from 1st October 2023 to 30th September 2024. Their efforts are greatly appreciated.

**NAME**

Abe, Yoshinari

Akechi, Tatsuo

Akizuki, Nobuya

Asada, Takahiro

Berdida, Daniel Joseph E.

Boku, Shuken

Chen, Chong

Chiba, Rie

Chien‐Chang Wu, Kevin

Ducharme, Simon

Enokido, Masanori

Escera, Carles

Eto, Nobuaki

Ferrao, Ygor Arzeno

Frank, Adam C.

Fujisawa, Daisuke

Fujishiro, Hiroshige

Fujita, Hirokazu

Fujita, Junichi

Fujiwara, Takeo

Fukao, Kenjiro

Fukao, Takashi

Fukui, Eriko

Fukushima, Haruko

Funatogawa, Tomoyuki

Funayama, Michitaka

Ghaderi, Ata

Hashimoto, Kenji

Hashimoto, Naoki

Hashimoto, Nozomu

Hatta, Kotaro

Hayasaka, Tomonari

Hayashi, Naoki

Hayashi, Wakaho

Higashi, Shinji

Hirai, Nobuhide

Hirata, Takashi

Hiromichi, Inaba

Ho, Roger

Hori, Arinobu

Hori, Hikaru

Ichihashi, Masanori

Iga, Junichi

Ikeda, Shunichiro

Imai, Ayu

Imai, Hissei

Imamura, Akira

Inada, Ken

Inoue, Shinichiro

Inoue, Takeshi

Iriki, Akihisa

Ishida, Takuto

Ishigaki, Takuma

Ishii, Ryouhei

Ishitobi, Makoto

Isobe, Masanori

Ito, Masanobu

Iwasa, Hajime

Iwata, Kazuhiko

Iwata, Masaaki

Iwata, Yusuke

Kageyama, Takayuki

Kamikubo, Atsushi

Kanazawa, Tetsufumi

Kaneko, Hitoshi

Katayama, Munenori

Kato, Hideo

Kato, Takahiro

Katsumata, Yotaro

Katsuta, Narimasa

Kavalidou, Katerina

Kawabe, Kentaro

Kawai, Hiroki

Kawashima, Yoshitaka

Kellner, Charles H.

Kenzaka, Tsuneaki

Kikuchi, Saya

Kindler, Jochen

Kishi, Yasuhiro

Knouse, Laura

Kobayashi, Daiki

Kobayashi, Masayoshi

Kobayashi, Ryota

Kobayashi, Toshiyuki

Koike, Shinsuke

Komatsu, Yuki

Kondo, Masaki

Kondo, Shinsuke

Kosaka, Hirotaka

Koshiyama, Daisuke

Kota, Munetsugu

Kubota, Manabu

Kumazaki, Tsutomu

Kunii, Yasuto

Kuramochi, Izumi

Kuriiyama, Hiroko

Lin, Chung‐Ying

Lui, Forshing

Machado, Andreia

Manabe, Yuta

Markowitz, John C.

Mastumoto, Yoshihiro

Matar, Elie

Matsubara, Toshio

Matsuki, Tasuku

Matsuzaka, Yusuke

Michie, Patricia

Misawa, Fuminari

Miyawaki, Dai

Mizuno, Eriko

Monden, Yukifumi

Monji, Akira

Morimoto, Takafumi

Morishima, Ryo

Moritz, Steffen

Mukai, Keiichiro

Murakami, Ryu

Murase, Yuji

Nakagami, Yukako

Nakanishi, Miharu

Nakano, Haruko

Narita, Hisashi

Negoro, Hideki

Nemoto, Kiyotaka

Nishida, Keiichiro

Nishimura, Tomoko

Nishizono‐Maher, Aya

Noda, Masataka

Noda, Yoshihiro

Nogimura, Akane

Noma, Shun'ichi

Ochi, Shinichiro

Ogata, Haruhiko

Ogawa, Yusuke

Ohashi, Kei

Okada Takashi

Okajima, Yoshiro

Okamoto, Yuri

Okubo, Ryo

Okuyama, Junko

Onitsuka, Takatoshi

Ono, Kazuya

Ota, Haruhisa

Ota, Toyosaku

Otani, Kyohei

Otsuka, Kotaro

Oya, Yoshio

Pijnenburg, Yolande

Rybakowski, Janusz

Sakamaki, Masanori

Sakamoto, Shinji

Sakamoto, Yui

Sakata, Masatsugu

Sakurai, Hitoshi

Sasabayashi, Daiki

Sasaki, Natsu

Sasaki, Tsuyoshi

Sato, Daisuke

Shigemura, Jun

Shima, Masayuki

Shimizu, Kanako

Shinba, Toshikazu

Shinohara, Kiyomi

So, Ryuhei

Sone, Daichi

Sun, Weiyi

Suwa, Taro

Suzuki, Keisuke

Suzuki, Masahiro

Tachikawa, Hirokazu

Tajika, Aran

Takagi, Shunsuke

Takahashi, Tomohisa

Takahashi, Tsutomu

Takano, Harumasa

Takeda, Takashi

Takekita, Yoshiteru

Takeshima, Masahiro

Takeshima, Tadashi

Tanaka, Satoshi

Tanaka, Shinichiro

Tani, Hideaki

Tateno, Amane

Tateno, Masaru

Terao, Takeshi

Terauchi, Masakazu

Terui‐Honma, Toshihiro

Terui, Toshihiro

Toda, Hiroyuki

Tomiyama, Hirofumi

Toyomaki, Atsuhito

Tsoh, Janice

Tsuboi, Takashi

Tsuchiya, Kenji

Tsujii, Noa

Tzeng, Nian‐sheng

Uchida, Hiroyuki

Uchida, Sunao

Uchiyama, makoto

Vinberg, Maj

Watanabe, Satsuki

Wright, Hayley

Yada, Yuji

Yamada, Hisashi

Yamada, Kazuo

Yamada, Yuto

Yamaguchi, Sosei

Yamamoto, Akira

Yamamuro, Kazuhiko

Yamauchi, Takahira

Yamauchi, Tsuneo

Yanagawa, Youichi

Yasuda, Kazuyuki

Yasui‐Furukori, Noiro

Yoshida, Kazunari

Yoshimura, Masafumi

Yoshimura, Reiji

Yoshino, Yuta

Yoshioka, Eiji

Young, Allan

Zhang, Shujing

